# Value of radiotherapy in addition to esophagectomy for stage II and III thoracic esophageal squamous cell carcinoma: Analysis of surveillance, epidemiology, and end results database

**DOI:** 10.1002/cam4.1731

**Published:** 2018-12-18

**Authors:** Jing Yu, Wen Ouyang, Yi Li, Jing Hu, Yu Xu, Yongchang Wei, Zhengkai Liao, Yu Liu, Junhong Zhang, Conghua Xie

**Affiliations:** ^1^ Department of Radiation and Medical Oncology Zhongnan Hospital of Wuhan University Wuhan China; ^2^ Department of Radiation Oncology Memorial Sloan Kettering Cancer Center New York New York

**Keywords:** Multimodality treatments, postoperative radiotherapy, preoperative radiotherapy, squamous cell carcinoma, thoracic esophageal carcinoma

## Abstract

To determine the value of radiotherapy in addition to esophagectomy for stage II and III TESCC. We searched the Surveillance, Epidemiology, and End Results (SEER) database for all cases of stage II‐III TESCC. Patients were grouped as those receiving pre‐ or postoperative radiotherapy plus esophagectomy and those receiving esophagectomy alone. Overall survival (OS) and cancer‐specific survival (CSS) were compared between the groups. Among the 3292 patients, multimodality treatments (pre‐ or postoperative radiotherapy plus surgery) were more effective than surgery alone (5‐year, OS: 17.3% vs 7.9%; *P *<* *0.001; CSS: 51.8% vs 34.9%; *P *<* *0.001). Among the patients receiving multimodality treatments, multivariate analyses revealed stage to be the most significant prognostic factor for OS (II vs III, HR, 0.726; *P *<* *0.001), but the sequence of radiotherapy and surgery was only of the marginal significance (pre‐ vs postoperative, HR, 0.875; *P *=* *0.093). Preoperative radiotherapy provided significantly better survival than postoperative radiotherapy in stage III disease (5‐year, OS: 13.0% vs 11.0%, *P *<* *0.04; CSS: 49.2% vs 31.7%, *P *<* *0.003), but not in stage II disease (5‐year OS: 23.5% vs 21.0%, *P *=* *0.519; CSS: 62.0% vs 53.4%, *P *=* *0.075). Radiotherapy in addition to esophagectomy provides better outcomes than esophagectomy alone for in stage II‐III TESCC. Preoperative radiotherapy followed by surgery appears to be the optimal treatment strategy in stage III TESCC.

## 1 INTRODUCTION

To date, there have been about 16 940 new occurrences of esophageal cancer in the United States each year, with approximately 15 690 deaths.^1^ In some endemic areas, for example, in China, esophageal cancer is the fourth leading cause of cancer‐related deaths.^2^

At presentation, more than one‐third of esophageal cancer patients have stage II or III disease.^3^ Multimodality treatments, including different combinations of radiotherapy and various chemotherapy regimens, may improve overall prognosis.^4^, ^5^, ^6^, ^7^, ^8^ Currently, preoperative chemoradiation followed by surgery is the most preferred treatment for stage II‐III thoracic esophageal squamous cell carcinoma (TESCC) of the middle or distal third.^9^, ^10^, ^11^ However, because of the pathological heterogeneity of patients enrolled in different studies, the survival benefit provided by preoperative chemoradiation has not yet been definitely established.^4^, ^12^

Unlike preoperative radiotherapy, postoperative radiotherapy is based on the precise pathological stage and allows delivery of higher doses to high‐risk volumes and thus reduces toxicities. These advantages are also likely to result in long‐term survival benefits over esophagectomy alone in TESCC patient.^13^, ^14^, ^15^ Thus, postoperative radiotherapy is regarded as an important treatment choice in China. However, previous studies investigating the efficacy of postoperative radiotherapy in TESCC have all been single‐institution studies and enrolled patients with various disease stages; the results therefore may not be generalizable to all TESCC patients.^13^, ^14^, ^15^ Furthermore, there have been no well‐designed prospective studies comparing the efficacy of preoperative vs postoperative radiotherapy in TESCC. Currently, available evidence is mainly from a subgroup analysis in the Chemoradiotherapy for Oesophageal Cancer followed by Surgery Study (CROSS). We therefore used the Surveillance, Epidemiology, and End Results (SEER) database to comprehensively analyze the outcomes from the different treatments to identify the optimal treatment strategy for guiding our clinical practice.

## 2 MATERIALS AND METHODS

### 2.1 SEER database

We searched the SEER linked database for data on patients diagnosed with TESCC between 2004 and 2014. The SEER cancer registries contain information on patient demographics, tumor characteristics, primary tumor sites, disease stages, first treatment course, and follow‐up. The SEER data are available for public use and include hospital, physician, outpatient, home health, and hospice data.

### 2.2 Patient selection

We used SEER*‐Stat version 8.3.4 software to extract data from the SEER database. Patients diagnosed with primary ESCC (SEER cancer site code: 27.0; SEER histology codes: 8052, 8070 to 8078, 8053, 8083, and 8084) in American Joint Committee on Cancer (AJCC) stages II to III were included in our study. As the AJCC stage 7th for esophageal cancer was published in 2009. Therefore, in our study, AJCC stage 6th was used for the patients diagnosed between 2004 and 2009, and stage 7th was applied for the cases diagnosed after 2010. To eliminate the influence of perioperative mortality, we included only patients who survived for ≥1 month after treatment completion.

Patients who were diagnosed with primary cervical ESCC (SEER cancer site code: C18.0) were excluded, because definitive chemoradiation is the most acceptable treatment for this subgroup of patients. All the patients needed to receive chemotherapy and those with unknown chemotherapy status were excluded. To eliminate bias resulting from differences in radiotherapy techniques, we only evaluated patients who underwent external beam radiation therapy. Finally, our study sample size was 3292 patients (Appendix S1).

### 2.3 Variables

The primary endpoints were 5‐year OS and cancer‐specific survival (CSS). OS was defined as the time from diagnosis to death from any cause. CSS was defined as the time from diagnosis to death from TESCC. The data extracted for this study included age, sex, race, pathologic grade, stage (based on AJCC criteria), primary tumor site, total number of lesions, and treatment strategies (i.e., preoperative radiotherapy + surgery vs postoperative radiotherapy + surgery vs surgery alone).

### 2.4 Statistical analysis

We compared the frequency of all patient‐ and disease‐related categorical variables between the different treatment strategies (preoperative radiotherapy + surgery vs postoperative radiotherapy + surgery vs surgery alone) using the Pearson chi‐square test. The 5‐year OS and CSS were assessed using the Kaplan‐Meier method and compared using the log‐rank test. Multivariate Cox proportional hazards analysis was performed to examine the potential prognostic value of the treatment strategies after adjustments for patient‐ and disease‐related covariates. The analyses were carried out using SPSS version 20.0 (IBM Corp., Armonk, NY, USA). Statistical significance was set at *P *≤* *0.05.

## 3 RESULTS

### 3.1 Patient characteristics

Among the 3292 TESCC patients, 2422 (73.5%) received surgery alone, 641 (19.5%) received preoperative radiotherapy + surgery, and 299 (7.0%) received surgery + postoperative radiotherapy. Age, sex, ethnicity, pathological grade, primary disease site, and total number of lesions significantly differed among the three treatment strategy groups (*P *<* *0.001). The proportion of young patients (<60 years) was significantly higher in the group receiving multimodality treatments (pre‐ or postoperative radiotherapy plus surgery) than in the group receiving surgery alone (*P *<* *0.001). The proportions of patients receiving the three different treatments were comparable in those with stage II and stage III disease (*P *<* *0.262). Table 1 lists the patient characteristics.

**Table 1:** Clinical characteristics of Patients undergoing resection for stage II and III thoracic esophageal squamous cell carcinoma

Variables	Patients no. (%)			
	Preoperative RT (N = 641)	Postoperative RT (N = 229)	Surgery alone (N = 2422)	*P*
Age (year)				
≤60	280 (43.7)	95 (41.5)	690 (28.5)	<0.001
>60	361 (56.3)	134 (58.5)	1732 (71.5)	
Sex				
Male	410 (64.0)	131 (57.2)	1605 (66.3)	<0.017
Female	231 (36.0)	98 (42.8)	817 (33.7)	
Race				
White	474 (73.9)	150 (65.5)	1521 (62.8)	<0.001
Other (Black/American Indian/AK Native, Asian/Pacific Islander)	167 (26.1)	79 (34.5)	901 (37.2)	
Grade				
Well differentiated/moderately differentiated	331 (51.6)	123 (53.7)	1142 (47.2)	<0.034
Poorly differentiated/undifferentiated	310 (48.4)	106 (46.3)	1280 (52.8)	
Stage				
II	307 (47.9)	97 (42.2)	1088 (44.9)	0.262
III	334 (52.1)	132 (57.8)	1334 (55.1)	
Primary site				
Upper or middle third of esophagus	342 (53.4)	138 (60.3)	1551 (64.0)	<0.001
Other (lower third of esophagus or NOS)	299 (46.6)	91 (39.7)	871 (36.0)	
Total number of lesions				
1	514 (80.2)	172 (75.1)	1699 (70.1)	<0.001
>1	127 (19.8)	57 (24.9)	723 (29.9)	

RT, radiotherapy.

### 3.2 Radiotherapy combined with surgery improved survival

**Figure 1:**
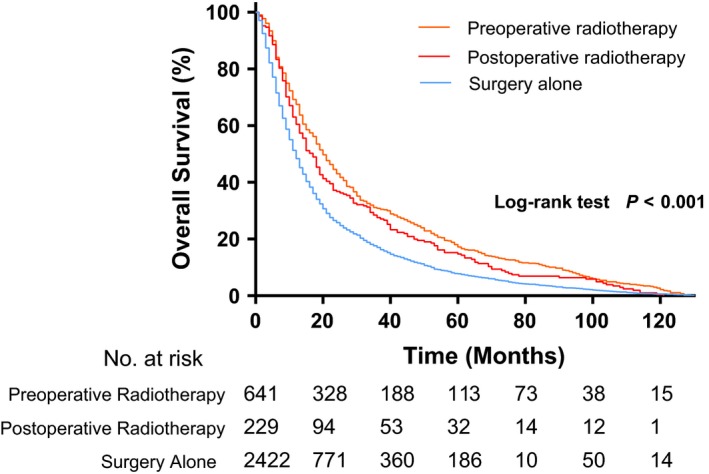
Comparison of OS among the preoperative radiotherapy + surgery, postoperative radiotherapy + surgery, and surgery‐alone groups. OS, overall survival

In the whole cohort, univariate analysis showed age, sex, ethnicity, stage, total lesion number, and treatment strategy to be significantly associated with OS (Appendix S2). The multimodality treatment (pre‐ or postoperative radiotherapy plus surgery) provided an additional survival benefit to the surgery‐alone group (5‐year, OS: 17.3% vs 7.9%; *P *<* *0.001). Preoperative radiotherapy plus surgery seemed to have the most favorable 5‐year OS. (Figure 1).

**Figure 2:**
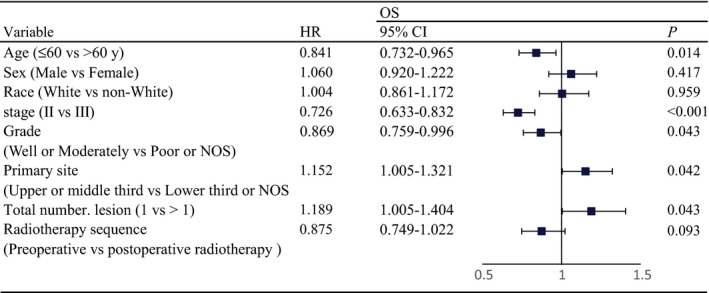
Multivariate analysis and forest plots indicating the independent prognostic factors of OS in TESCC patients receiving multimodality treatments with radiotherapy and surgery. OS, overall survival; TESCC, thoracic esophageal squamous cell carcinoma; HR, hazard ratio; CI, confidence interval

To define the optimal treatment strategy, we next assessed the efficacy of different sequences of radiotherapy and surgery (i.e., preoperative radiotherapy plus surgery vs postoperative radiotherapy plus surgery) in the multimodality treatment group. Multivariate analysis revealed that age, stage, pathological grade, primary site, and total number of lesions were all independent prognostic factors (*P *<* *0.05). The sequence of radiotherapy and surgery was only of the marginal significance (pre‐ vs postoperative radiotherapy, HR, 0.875; 95% CI, 0.749 to 1.022; *P *=* *0.093; Figure 2).

### 3.3 Survival is similar with preoperative radiotherapy and postoperative radiotherapy in stage II disease

**Figure 3:**
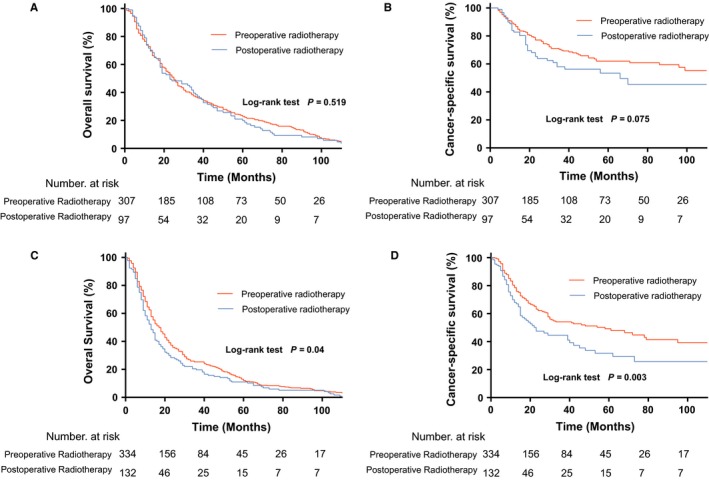
Comparison of OS and CSS in patients treated with preoperative radiotherapy + surgery vs postoperative radiotherapy + surgery for stage II (A, B) and stage II TESCC (C, D). OS, overall survival; CSS, cancer‐specific survival; TESCC, thoracic esophageal squamous cell carcinoma

Because stage was found to be the most significant prognostic factor (II vs III; HR, 0.726, 95% CI, 0.633 to 0.832; *P *<* *0.001; Figure 2), we stratified the patients into two different risk groups for further evaluation. In stage II patients, all clinical characteristics were comparable between preoperative radiotherapy and postoperative radiotherapy groups (Table 2). The 5‐year OS did not significantly differ between the two groups (23.5% vs 21.0%; *P *=* *0.519; Figure 3A). To eliminate the influence of noncancer deaths, we compared the 5‐year CSS between the two treatment groups; however, 5‐year CSS was comparable between the preoperative and postoperative radiotherapy groups (62.0% vs 53.4%; *P *=* *0.075; Figure 3B).

**Table 2:** Clinical characteristics of stage II and III thoracic esophageal squamous cell carcinoma patients

Variables	II stage patients (N = 404)			III stage patients (N = 466)		
	Pre‐RT+S patients (%)	Post‐RT+S patients (%)	*P*	Pre‐RT+S patients (%)	Post‐RT+S patients (%)	*P*
Total	307	97		334	132	
Age (year)						
≤60	140 (45.6%)	35 (36.1%)	0.099	140 (41.9%)	60 (45.5%)	0.487
>60	167 (54.3%)	62 (63.9%)		194 (58.1%)	72 (54.5%)	
Sex						
Male	195 (63.5%)	57 (58.8%)	0.399	215 (63.4%)	74 (56.0%)	0.096
Female	112 (36.5%)	40 (41.2%)		119 (36.6%)	58 (44.0%)	
Race						
White	234 (76.2%)	63 (64.9%)	0.09	240 (71.9%)	87 (65.9%)	0.448
Non‐White	73 (23.8%)	34 (35.1%)		94 (28.1%)	45 (34.1%)	
Grade						
Well/moderately differentiated	167 (54.4%)	55 (56.7%)	0.691	164 (49.1%)	68 (51.5%)	0.639
Poorly differentiated/undifferentiated	140 (45.6%)	42 (43.3%)		170 (50.8%)	64 (48.4%)	
Primary site						
Upper/middle third	175 (57.0%)	61 (62.9%)	0.305	167 (50.0%)	77 (58.3%)	0.105
Low third/not specified	132 (43.0%)	36 (37.1%)		167 (50.0%)	55 (41.6%)	
Total number of lesions						
1	241 (78.5%)	74 (76.2%)	0.647	273 (81.7%)	98 (74.2%)	0.07
>1	66 (21.5%)	23 (23.7%)		61 (18.3%)	34 (25.8%)	

RT, radiotherapy.

### 3.4 Preoperative radiotherapy improves survival in stage III disease

Among stage III disease patients, the clinical characteristics were comparable between the preoperative radiotherapy and postoperative radiotherapy groups. The 5‐year OS was significantly better with preoperative radiotherapy than with postoperative radiotherapy (13.0% vs 11.0%; *P *<* *0.04, Figure 3C). The 5‐year CSS was also significantly better in the preoperative radiotherapy group (49.2% vs 31.7%; *P *<* *0.003; Figure 3D).

## 4 DISCUSSION

The present study, based on data obtained during a 10‐year period (2004‐2014), found that radiotherapy combined with surgery provided better OS than surgery alone in stage II and III TESCC. Further, the study showed that although survival outcomes were comparable with preoperative radiotherapy and postoperative radiotherapy in stage II TESCC, preoperative radiotherapy provided significantly improved survival in stage III disease. To our knowledge, this is the one of very few studies evaluating the efficacy of pre‐ and postoperative radiotherapy plus surgery in TESCC patients during the modern era.

At present, preoperative chemoradiation combined with surgery is the preferred recommended treatment for locally advanced esophageal cancer. A series of meta‐analyses has reported that compared with surgery alone, preoperative chemoradiation followed by surgery increases the locoregional control rate and reduces the risk of death in patients with resectable esophageal cancer (mortality risk decline: squamous cell carcinoma, 20%; adenocarcinoma, 25%).^16^, ^17^, ^18^ The landmark CROSS study found that preoperative chemoradiation not only improved locoregional control but also provided better long‐term survival outcomes than surgery alone in both squamous cell carcinoma and adenocarcinoma.^11^ However, some researchers have argued that the relatively low proportion of squamous cell carcinoma patients in the CROSS population makes it difficult to extrapolate the conclusions to both pathological types. In this study, we confirmed that combined preoperative radiotherapy and surgery provided an approximately 10% absolute survival benefit as compared to surgery alone. This suggests that preoperative radiotherapy combined with surgery should be recommended in patients with stage II and III TESCC.

However, the survival outcomes of our study are inferior to the results of the CROSS study (5‐year OS: 18.0% vs 47%) but comparable to those from French and German studies (2‐year OS: 44.6% vs 30%‐34%).^12^, ^19^ Considering that age is a significant prognostic factor for OS (Appendix S2), the higher proportion of elderly patients included in our study may account for the poorer survival outcomes. Additionally, in contrast to the CROSS study, our study included patients with more locally advanced disease, with 580 (17.6%) T4 patients being entered in our analysis; this may also explain the relatively inferior survival outcomes.

The efficacy of postoperative radiotherapy in TESCC has not been demonstrated using randomized trials. Most studies have reported that surgery followed by postoperative radiotherapy provided an additional survival benefit predominantly to patients with stage III esophageal carcinoma.^20^, ^21^, ^22^, ^23^ However, some clinicians regard postoperative radiotherapy as crucial additional treatment for patients with stage II TESCC.^24^, ^25^ To clarify these conflicting findings, we reassessed the role of postoperative radiotherapy in our cohort and found that postoperative radiotherapy was more effective than surgery alone. Our data were collected from 2004 to 2014, during which modern, precise radiotherapy techniques were increasingly used. Therefore, the study demonstrates that the use of highly efficient postoperative radiotherapy techniques with low treatment‐related toxicities can improve outcomes in both stage II and stage III TESCC.

After confirming the significant survival advantage provided by combined‐modality treatments (pre‐ or postoperative radiotherapy plus surgery) over surgery alone, we compared the effects of pre‐ and postoperative radiotherapy in this combined‐modality treatment subgroup. In this cohort, stage was the most significant prognostic factor. The order of treatment (radiotherapy before or after surgery) was only of marginal significance. When we examined stage II and stage III disease patients separately, we found that in low‐risk stage II disease, the outcomes were similar with preoperative and postoperative radiotherapy; however, in high‐risk stage III TESCC, preoperative radiotherapy provided better outcomes than postoperative radiotherapy. Although (as Table 1 shows) the stage of TESCC (II vs III) did not affect the treatment choice of clinicians, preoperative radiotherapy plus surgery might increase the opportunities for radical resection in high‐risk stage III TESCC. We speculate that this might be the explanation for the additional survival benefit of preoperative radiotherapy.

A previous study has reported that preoperative chemoradiation plus surgery was more likely to improve OS in TESCC with clinical T3/4 stage or tumor size >5 cm than postoperative chemoradiation.^26^ However, this conclusion was based on analysis of a relatively small subgroup and, moreover, the authors did not adjust for the influence of other variables such as age or lymph node status. Therefore, our results, based on analysis of risk‐adapted therapy and different stages, seem more reasonable.

This study has some limitations. The retrospective nature of the study and the significant differences in clinical characteristics among the treatment groups may result in selective bias. To decrease the influence of selective bias, we used multivariate analysis to confirm the potential prognostic value of the different treatment strategies after adjustments the covariates. In addition, after stratification by stages, all relevant variables were comparable, and univariate analysis was sufficient to assess the efficacy between pre‐ and postoperative radiotherapy. Moreover, our findings are based on a relatively large sample, which might be more representative of the data in the real world. Another limitation is that we did not analyze disease‐free survival, recurrence patterns, and safety. Further prospective studies are needed to validate these results.

## CONFLICT OF INTEREST

None declared.
